# Mutational analysis of the *RB1* gene in Moroccan patients with retinoblastoma

**Published:** 2011-12-30

**Authors:** Omar Abidi, Sara Knari, Hajar Sefri, Majida Charif, Audrey Senechal, Christian Hamel, Hassan Rouba, Khalid Zaghloul, Asmaa El Kettani, Guy Lenaers, Abdelhamid Barakat

**Affiliations:** 1 Laboratoire de Génétique Humaine et Biologie Moléculaire, Institut Pasteur du Maroc, Casablanca, Morocco; 2 Service d’Ophtalmologie pédiatrique, Hôpital 20 Août, CHU de Casablanca, Morocco; 3 Institut des Neurosciences de Montpellier, Génétique et thérapie des cécités rétiniennes et neuropathies optiques héréditaires, INSERM U-1051, Université de Montpellier Sud de France, Montpellier, France

## Abstract

**Purpose:**

Retinoblastoma (RB), the most common intraocular tumor occurring in infancy and early childhood, is most often related to mutations in the *RB1* gene. In this study, we screened the *RB1* germline mutations in 41 unrelated Moroccan patients with retinoblastoma, 25 heritable cases, and 16 sporadic unilateral cases.

**Methods:**

After complete ophthalmic examinations were performed and consent obtained, DNA was extracted from peripheral blood, and screening of *RB1* mutations was performed with PCR direct sequencing of the promoter and the 27 coding exons of the *RB1* gene.

**Results:**

We identified ten germline mutations in 10/41 (24.39%) unrelated patients, among which three had not been previously reported. The mutation detection rate was 40% (10/25) in the heritable cases and 0% (0/16) in the sporadic unilateral cases. Of these mutations, six were nonsense, and three were frameshifts, all associated with severe phenotypes resulting in bilateral and multifocal tumors. One splice site mutation was found in a familial case associated with a low expressivity phenotype resulting in unilateral and unifocal tumors. Moreover, eight intronic variants were identified, three of which were novel.

**Conclusions:**

This first report of *RB1* gene screening in Moroccan patients with retinoblastoma shows a comparable mutational spectrum to those reported previously, which has evident importance for managing patients with retinoblastoma and their families.

## Introduction

Retinoblastoma (RB, OMIM 180200], the most frequent childhood intraocular tumor, is caused by mutations in the *RB1* gene. The tumor arising from embryonic retinal cells occurs in 1:15,000 to 1:20,000 live births, with no bias in the sex ratio [1]. In Morocco, the incidence of RB was estimated at about 50 new cases annually [2]. Worldwide, approximately 40% of RB cases are heritable cases. The predisposition to develop RB is inherited as an autosomal dominant trait, but mutations in both alleles are necessary to cause the disease [3]. Individuals carrying a germline mutation are predisposed within the first 2 years of life to multifocal bilateral tumors, and later in adulthood to bone and connective tissue tumors [4,5]. The germline mutations are associated with predisposition with a high penetrance (90% or more) to RB and most often inherited from an affected parent (hereditary familial RB) or acquired during gametogenesis or gestation (hereditary de novo) [3,6]. Nevertheless, low penetrance and variable expressivity of the disease have been described in some families presenting bilateral or unilateral RB, benign retinoma, or unaffected carriers [7–9]. Non-hereditary RB, representing 60% of cases, is unilateral and does not induce increased lifetime risk of non-ocular tumors. In these cases, mutations in both *RB1* alleles take place in a single retinal cell that will develop to form the tumor [1,10,11].

Mutations in the *RB1* gene are highly heterogeneous and scattered in the promoter and the 27 coding exons. Previous reports described a wide-ranging mutation detection rate, from 5.5% to 94.8%, according to the patient selection criteria (unilateral, bilateral, familial, and/or sporadic cases) and the screening techniques [12–19]. Single base substitutions represent the most frequent mutations, and among them, nonsense mutations predominate (Retinoblastoma genetics). The existence of genetic modifiers in RB was recently identified in the *MDM2* gene as a modulator of disease severity [20,21].

To identify the spectrum and the effect of germline mutations and to provide accurate genetic counseling, we performed a genetic analysis of the *RB1* gene in a Moroccan retinoblastoma cohort.

## Methods

### Patients

This study included 41 unrelated cases of RB with different clinical presentations, recruited between November 2009 and August 2010. Of these, 25 had heritable disease with 23 sporadic bilateral and two familial cases, and 16 had sporadic unilateral RB. The patients, 20 girls and 21 boys (sex ratio of 0.95) with age at diagnosis ranging from 2 to 36 months, were examined and treated at the pediatric ophthalmology department of the 20 Aout Hospital, CHU of Casablanca, Morocco. Clinical features (tumor laterality, type and number of tumor foci) were diagnosed for each patient and associated with a questionnaire. Blood samples were obtained in standard EDTA blood collection tubes from patients and available relatives after individual written consent for genetic analysis was obtained, in accordance with the Declaration of Helsinki. The samples were stored at −20 °C until nucleic acid extraction.

### Molecular analysis

DNA was extracted from peripheral blood using the phenol/chloroform method [22]. Screening of germline mutations in the *RB1* gene was performed with PCR-directed sequencing of the promoter and the 27 coding exons, and their flanking intronic regions, using the primers described by Abouzeid et al. [8]. PCR reactions were performed in a thermal cycler (ABI 2700), in a total volume of 15 µl containing 25–50 ng of genomic DNA, 3 picomoles of each primer, and 7.5 µl of master mix 2× (AmpliTaq Gold 360 Master Mix; Applied Biosystems, Foster City, CA). Reactions were performed for 35 cycles of 94 °C for 1 min, annealing at the specific temperature for 1 min, 72 °C for 1 min, and a final extension step at 72 °C for 7 min. After the unincorporated deoxynucleoside triphosphates and primers were removed using Exonuclease I and shrimp alkaline phosphatase (ExoSAP-IT PCR Purification Kit, GE Healthcare, UK), the PCR products were directly sequenced using the ABI BigDye Terminator v 3.1 Sequencing Standard Kit (Applied Biosystems, Foster City, CA) and run on an ABI 3130 Genetic Analyzer. The sequence data were analyzed by comparison to the consensus sequence of the RB1 gene (GenBank L11910.1) using ABI SeqScape v2.5 software. Additional information about mutations and variants was obtained from the RB1 gene database.

## Results

Out of the 41 RB patients tested for *RB1* germline mutations, we found 25 (61%) heritable cases with 23 sporadic bilateral and two familial cases and 16 (39%) nonhereditary cases, sporadic unilateral. The mean age at diagnosis was 10.54 months for bilateral patients and 17 months for unilateral patients. In total, we identified ten causative RB1 mutations in ten out of 41 patients (global mutation rate of 24.39%; Table 1). Among these patients, nine had sporadic bilateral RB, and one had familial unilateral RB. Therefore, the detection rate of *RB1* mutations was 40% (10/25) in heritable cases and 0% (0/16) in sporadic unilateral cases. Three mutations (30%) were novel, and seven (70%) had been previously reported (*RB1* gene database). Seven mutations were single-base substitutions leading to six nonsense (p.Arg358X, p.Arg455X, p.Arg552X, p.Tyr651X, p. Lys652X, p.Arg787X) amino acid changes and one splice site mutation (c.719–2A>G) in intron 7. The three other mutations were small deletions of one or two base pairs causing a frameshift and premature termination (p.Ile124ArgfsX5, p.Ile214PhefsX4, p.Lys647PhefsX4) of the open reading frame. The analysis of the splice site mutation (c.719–2A>G) with Human Splicing Finder software showed that the consensus value of the wild-type splice acceptor site was decreased by 33.5%, suggesting a partial disruption of the normal splicing event.

**Table 1:** RB1 germline mutations identified in Moroccan patients with retinoblastoma

**Patient**	**g-position^a^**	**cDNA change^b^**	**Protein**	**Location**	**Laterality**	**Occurrences^c^**
RB3	g.153347A>T	c.1954A>T	p.Lys652X	Exon 19	Bilateral	Yes (1)
RB4	g.39552_39553delTA	c.371_372delTA	p.Ile124ArgfsX5	Exon 3	Bilateral	Yes (3)
RB5	g.162237C>T	c.2359C>T	p.Arg787X	Exon 23	Bilateral	Yes (62)
RB14	g.153332_153333delCT	c.1939_1940delCT	p.Lys647PhefsX4	Exon 19	Bilateral	Yes (4)
RB16	g.56885delA	c.640delA	p.Ile214PhefsX4	Exon 7	Bilateral	No
RB19	g.153346T>G	c.1953T>G	p.Tyr651>X	Exon 19	Bilateral	No
RB20, RB21	g.59649A>G	c.719–2A>G	Altered splicing	Intron 7	unilateral	No
RB26	g.78238C>T	c.1654C>T	p.Arg552X	Exon 17	Bilateral	Yes (63)
RB37	g.76460C>T	c.1363C>T	p.Arg455X	Exon 14	Bilateral	Yes (53)
RB39	g.65386C>T	c.1072C>T	p.Arg358X	Exon 11	Bilateral	Yes (60)

^a^position in *RB1* genomic sequence (GenBank accession number L11910.1) ^b^Nucleotide numbering reflects cDNA numbering with +1 corresponding to the A of the ATG translation initiation codon in the reference sequence (NM_000321.2). ^c^Number of occurrences (in parenthesis) in the literature based on the *RB1* gene database 2011.

**Figure 1:**
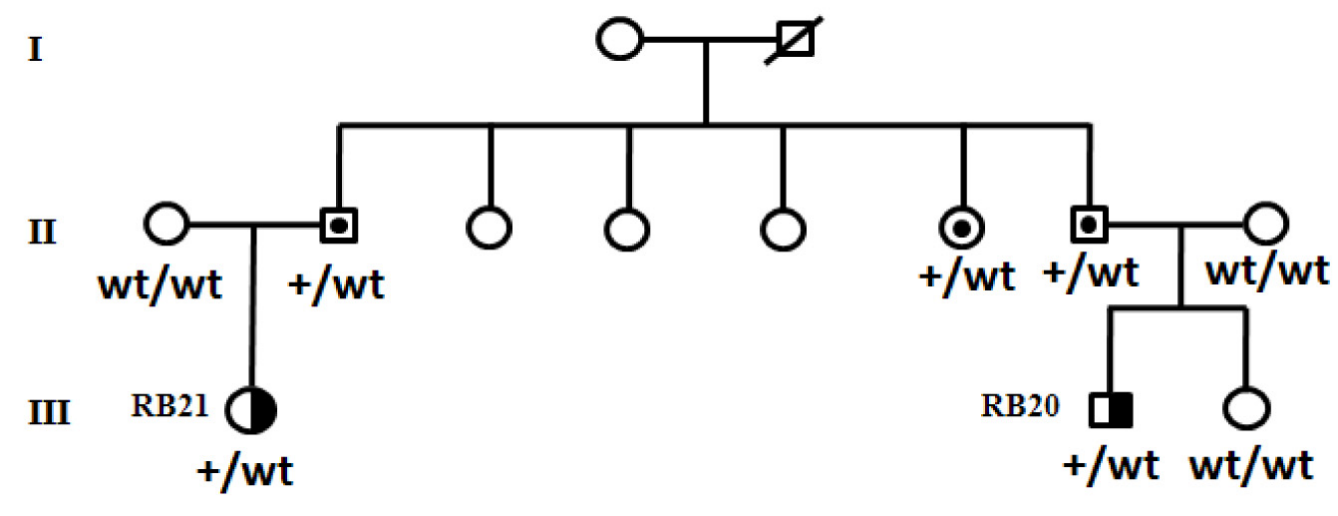
Segregation of the c.719–2A>G splice mutation in the RB20 and RB21 families associated with unilateral retinoblastoma. The genotype is provided for the tested members as wt/+ for heterozygous carriers and wt/wt for homozygous relatives. Half-filled symbols: affected unilateral patients; dotted symbols: unaffected carriers.

Additional genetic analysis among members of the RB4, RB5, RB14, and RB37 families revealed four bilateral sporadic cases, for whom the causative mutations were absent in the parents, suggesting that these mutations are de novo, potentially expressed in a germinal mosaicism state in one of the asymptomatic parents. For the RB14 family, the mutation was also absent in the two siblings; in one, the genetic analysis was performed on umbilical cord blood. The analysis of the RB20 and RB21 cases belonging to the same family showed the presence of the same causative splice site mutation (c.719–2A>G) in the patients, which was associated with unilateral and unifocal RB. The transmission analysis (Figure 1) of this mutation showed that their fathers and other family members were healthy carriers of this mutation, without any clinical symptoms.

In addition, the genetic analysis showed eight intronic *RB1* changes not predicted to be pathogen variants (Table 2). Of these, seven were single-base substitutions: g.39598A>G, g.39606T>C and g.39573T>C (intron 3), g.42068G>T (intron 4), g.59643T>C (intron 7), g.73724A>G (intron 12), and g.150191A>G (intron 19); and one deletion of seven bases in the intron 12 (g.73734_73740delCTGTTTT). The new changes (g.59643T>C, g.150191A>G, and g.73734_73740delCTGTTTT) were evaluated with Human Splice Finder and were not predicted to have an impact on splicing.

**Table 2:** RB1 intronic variants detected in Moroccan patients with retinoblastoma

**g-position**	**cDNA change**	**Location**	**Occurrences^b^**
g.39598A>G	c.380+37A>G	Intron 3	Yes (2)
g.39606T>C	c.380+45C>T	Intron 3	Yes (3)
g.39573T>C	c.380+12T>C	Intron 3	Yes (2)
g.42068G>T	c.500+23G>T	Intron 4	Yes (2)
g.59643T>C^a^	c.719–8T>C	Intron 7	No
g.73724A>G	c.1216–29A>G	Intron 12	Yes (2)
g.73734_73740delCTGTTTTa	c.1216–19_-13del	Intron12	No
g.150191A>G^a^	c.1814+74A>G	Intron 19	No

^a^New variants were tested for pathogenicity by using the Human Splicing Finder software. ^b^Number of occurrences (in parenthesis) in the literature based on the RB1 gene database 2011.

## Discussion

This is the first report of *RB1* mutational screening in Moroccan patients with RB. The screening of *RB1* germline mutations in 41 patients identified ten different mutations. The global mutation detection rate was 24.39% (ten out of 41 patients) with 40% (10/25) in the heritable cases and 0% (0/16) in the sporadic unilateral cases. Previous reports described a high heterogeneity of the detection rate ranging from 19% to 72% (Table 3). [8,14,16,17,19,23–31]. This rate increases for heritable cases, with a maximum of 94.8% detected by a recent study performed on 1,024 patients [19]. For sporadic unilateral cases, researchers have postulated that about 10%–12% of cases are caused by germline mutations [1]. In 90 sporadic unilateral probands of French origin, Houdayer et al. [14] found germline mutations in 5.5% of the cases. However, based on a small selected sample of 21 Indian unilateral patients, mutations were detected in 23.8% of cases [16]. In addition, on a widely selected sample of 385 unilateral cases, Rushlow et al. [19] found 52 (13.5%) carriers of mutations in blood. This heterogeneity of the mutation detection rate could mainly be explained by the technique diversity used and the size of the cohorts studied (Table 3). Indeed, some techniques [18,32] such as direct sequencing or denaturing high pressure liquid chromatography [14,16,24,30] are known as more efficient to detect point mutations. Others such as cytogenetic, fluorescent in situ hybridization, and quantitative multiplex PCR techniques are more efficient for detecting large gene rearrangements. Recently, Houdayer et al. (2011) [33] described a new method, called multiplex PCR/liquid chromatography, to screen for large deletions and duplications. The European Molecular Quality Network (EMQN) recommended combining two or more different methods to increase the sensitivity of *RB1* mutation detection. The mutation rate reported in the present study is similar to those found by other groups using screening techniques based on PCR sequencing (Table 3) [28,29]; however, higher detection rates have been reported when a combination of approaches was used [14,16,18,19]. Thus, other mutation screening technologies sensitive to short and large rearrangements should be used to maximize the detection rate in our cohort. In addition to the techniques used for detection analysis, other explanations could be provided to explain the high rate of cases negative for *RB1* mutations, especially for patients with heritable RB. In the patients who were *RB1* negative, 16 were unilateral sporadic cases, either bearing two somatic mutations or having an *RB1* mutation in a mosaic state. In the heritable cases, consisting of one familial and 14 bilateral sporadic presentations, the absence of mutation could be explained either by the inactivation of *RB1* through mutation in non-coding regions situated outside the explored sequences or by an epigenetic alteration, as bilateral sporadic cases are carriers of somatic mosaicism and cannot be detected in blood samples [16,19,27]. In addition, the large size of the *RB1* gene, the high degree of mutational heterogeneity of the disease (>900 *RB1* mutations), and the quality of the sequencing electropherograms could also have caused the high rate of negative cases in our study.

**Table 3:** Detection rate of RB1 germline mutations in patients with retinoblastoma reported from different countries

**Population**	**Method(s) used**	**No. of patients**	**Phenotype**	**Global rate^a^**	**% in F or SB^b^**	**% in SU^c^**	**% NS/FS mutations**	**% Splicing mutations**	**Reference**
Japan	SSCP, DHPLC, FISH	51	11 FB 4 FU 16 SB 20 SU	39	61	5	70	15	[23]
Germany	SSCP, HDA, sequencing	71	B or F	72	72	ND	88	11	[24]
Spain, Colombia and Cuba	Sequencing, microsatellite markers	107	11 FB 4 FU 49 SB 43 SU	50	67	20.9	58	23	[25]
China	SSCP	42	14 SB 28 SU	19	50	3.6	63	37	[26]
Italy	SSCP, sequencing, real-time PCR	35	7 FB 2FU 13 SB 13 SU	37	59	0	62	31	[27]
New Zeland	Sequencing MLPA, FISH, bisulphite method	20	1FB 7SB 12 SU	50	100	17	60	30	[17]
Europe, North America, Asia	QM-PCR, Sequencing, AS-PCR	1020	421 B 27 FU 572 SU	49	94	14.6	ND	ND	[19]
Switzerland	DHPLC, Sequencing, STR markers	65	7 F 30 SB 28 SU	45	70	10.7	68	27	[8]
India	QM-PCR RFLP, FG, Sequencing	74	53 B 4FU 17 SU	66	84	6	28.5	12.3	[16]
France	DHPLC, QMPSF	192	102 B or F 90 SU	46	81.5	5.5	51	26	[14]
Mexico	SSCP-Sequencing	48	21 B 27 U	19	ND	ND	31	ND	[28]
Argentina	Sequencing	21	6 FB 7 SB 8 SU	24	80	12.5	80	0	[29]
North America	Sequencing, RT, QSBA, LOH	180	85 B 10 FU 85 SU	50	88	7	ND	ND	[30]
Spain	Sequencing, RT–PCR	43	43 SB or F	67	67	ND	69	31	[31]
Morocco	Sequencing	41	1FB 1 FU 23 SB 16 SU	24	40	0	90	10	This study

NS/FS: Nonsense/frameshift mutations; SSCP Single-strand conformational polymorphism; DHPLC denaturing high performance liquid chromatography; FISH fluorescent in situ hybridization; HDA: Heteroduplex Analysis; MLPA: multiplex ligation probe amplification; QM-PCR: Quantitative Multiplex-PCR; QMPSF: Quantitative Multiplex PCR of short fluorescent fragments; AS-PCR: Allele specific-PCR; FG: Fluorescent genotyping; QSBA: Quantitative Southern Blot Analysis; LOH: Loss Of Heterozygocity; S: sporadic; F: familial; U: unilateral; B: bilateral; ND: not determined ^a^Detection rate of germline mutations in all patients with retinoblastoma ^b^Detection rate of germline mutations in heritable cases (sporadic bilateral or familial cases). ^c^Detection rate of germline mutations in sporadic unilateral cases.

While the frequency of mutations detected varies widely, the pattern of mutations is very similar in various populations, the most predominant categories being nonsense and frameshift mutations (Table 3). Our result is comparable to those reported in these studies with about 90% of mutations detected being nonsense or frameshift. In addition, the mutational spectrum found in our cohort does not differ from those reported previously, even if we found novel mutations, confirming that the mutations causing RB have occurred independently in the majority of cases in the *RB1* gene database.

On the other hand, the penetrance and expressivity of heritable retinoblastoma may be determined by the mutation type in *RB1*. Nonsense and frameshift mutations typically cause multifocal bilateral tumors due to the absence of the retinoblastoma protein, whereas intronic splice mutations are associated with incomplete penetrance and milder expressivity due to a residual function of the retinoblastoma polypeptide [7,18,24,34,35]. In our cohort, all patients exhibiting nonsense and frameshift mutations had bilateral multifocal tumors, while the two familial cases displaying the splice site mutation had unilateral unifocal disease. In addition, the mutation screening of other family members for this splicing mutation (Figure 1) detected three unaffected carriers suggesting low expressivity and penetrance of this mutation. Researchers have reported that the existence of low penetrance and variable expressivity of the disease in some families presenting either bilateral or unilateral affected patients, or unaffected carriers is related to the implication of modifier genes [7,20]. Recently, Castera et al. [21] demonstrated that the *MDM2* gene is the first modifier gene for retinoblastoma. In this study, the genotyping of the *MDM2-*SNP309T>G (rs2279744) polymorphism, assessed in 70 RB families (212 carriers of germline mutations and 114 relatives), revealed a significant association of the SNP309G allele with the presence of bilateral or unilateral tumors among mutation carriers reflecting a recessive contribution of *MDM2* to tumor susceptibility [21].

Finally, researchers have reported that the majority of patients with bilateral RB have sporadic disease with no familial transmission, which arises by de novo mutation of *RB1* either in the germline or embryo [16]. In our cohort, the parental screening of four probands with germline mutations indicated that these cases were associated with de novo mutations.

In conclusion, in spite of the technical difficulty of analysis, screening for the *RB1* gene remains an integral component of managing patients with retinoblastoma. This screening allows the prediction of risk in relatives of probands presenting germline mutations, early management and detection of tumors in predisposed individuals, and the avoidance of invasive and unnecessary examinations of non-carriers.
